# Supplementary material for: Minimum acceptable dietary intake among children aged 6–23 months in Ethiopia: A systematic review and meta-analysis
Source: PLoS One. 2023 Jun 29;18(6):e0287247. doi: 10.1371/journal.pone.0287247 (PMC10310018; doi:10.1371/journal.pone.0287247)
Supplement: S1 File — (DOCX) [file pone.0287247.s002.docx]

S2 File: Searching strategy for systematic review and meta-analysis on minimum acceptable diet among children aged 6-23 months in Ethiopia, 2022

| **Database** | **Example of searching strategy** |
| --- | --- |
| PubMed | ((((("minimum acceptable diet *") OR (meal frequency) OR (ITN)) AND ((((((("Prevalence"[All Fields]) OR ("intake*"[All Fields])) OR ("feeding"))) AND ((children aged 6-23 months)) AND ((((((ethiopian) OR (ethiopia)) OR ("southern Ethiopia")) OR ("northern Ethiopia")) OR ("eastern Ethiopia")) OR ("western Ethiopia")) |
| Google Scholar | " minimum acceptable diet " OR“meal frequency OR dietary diversity and utilization " " AND “children aged 6-23 months women” AND "Ethiopia" |
| AJOL | (“minimum acceptable diet” OR “meal frequency OR dietary diversity and “prevalence” AND Ethiopia |
| science direct | “minimum acceptable diet" AND “children aged 6-23 months” AND "Ethiopia” |
| Cochrane Library | ("minimum acceptable diet [MeSH]" OR meal frequency OR dietary diversity and utilization) AND children aged 6-23 months AND Ethiopia |
