# Supplementary material for: Minimum acceptable dietary intake among children aged 6–23 months in Ethiopia: A systematic review and meta-analysis
Source: PLoS One. 2023 Jun 29;18(6):e0287247. doi: 10.1371/journal.pone.0287247 (PMC10310018; doi:10.1371/journal.pone.0287247)
Supplement: Attachment — Submitted filename: Reviewer Comments_CB Witten_6 June 2022.docx [file pone.0287247.s003.docx]

Journal Name: PLOS ONE

Minimum Acceptable Dietary intake among Children Aged 6-23 Months in Ethiopia: a Systemic Review and Meta-Analysis

--Manuscript Draft—

Reviewer: Dr Chantell Witten

Well written and methodological sound manuscript. A few minor corrections:

Line 45: Better to say 1 in 4 children rather than one fourth of children

Line 81: Sentence needs restructuring to read as follows:- ‘This indicates that feeding of the minimum acceptable diet is a major problem in Ethiopia. You cannot say global problem when you have only presented data for Ethiopia

Line 170: Capital letters for Egger’s and Begg

Line 191: Define cases and Prevalence in your table. What cares and prevalence of what?

Line 235: restructure sentence to read ‘national Demographic and Health surveys in China, ….”

Line 237: remove ‘finding from’

Line 238: ‘in low- and middle-income countries…’

Line 234: heterogeneity without capital H

Line 256: Correction: higher prevalence of MAD was observed in studies conducted in urban settings compared to studies conducted in **rural** settings.

Line 265: restructure sentence to read ‘more likely to be fed the MAD standard…’

Line 268: delete after the end of the MDGs

Line 268-271: This sentence does not add any value to the discussion of IYCF or MAD. Or restructure to say how the new 2019 Nutrition programme is addressing IYCF or MAD.

Line 277: 1 in 4 children rather than ‘one fourth of children…’
