# Supplementary material for: Minimum acceptable dietary intake among children aged 6–23 months in Ethiopia: A systematic review and meta-analysis
Source: PLoS One. 2023 Jun 29;18(6):e0287247. doi: 10.1371/journal.pone.0287247 (PMC10310018; doi:10.1371/journal.pone.0287247)
Supplement: Attachment — Submitted filename: Response to reviewer.docx [file pone.0287247.s004.docx]

**July 8, 2022**

**Yogan Pillay, PhD
Academic Editor
PLOS ONE**

**RE: PONE-D-22-14108: Minimum Acceptable Dietary among Children Aged 6-23 Months in Ethiopia: a Systemic Review and Meta-analysis**

**Dear Dr. Yogan Pillay,**

We thank you for considering our manuscript and for arranging for it to be reviewed by reviewer. We have tried to address your comments and the comments / suggestions from the reviewer. We have also incorporated changes to reflect most of the suggestion provided by you and the reviewer. We have highlighted the changes within the manuscript please find for your kind consideration the following: In the Response to Reviewers, we copy each of the *comments* / *suggestions* and provide the **RESPONSE** hereunder. We also provide a marked-up copy of the manuscript that highlights changes made to the original version and this is uploaded as a separate file labelled “*Revised Manuscript with Track Changes*”. Finally, we provide an unmarked version of the revised manuscript without tracked changes and this is uploaded as a separate file labelled *'Manuscript*'.

We have been carefully through the peer review and have revised our paper accordingly. We feel that the paper is much improved as a result of this peer review process, and thank you for taking it to this stage. While hoping that these changes would meet with your favorable consideration, we hold ourselves at your entire disposition for any further information or other changes you might require. The authors welcome further constructive comments.

Sincerely yours!

Gizachew Ambaw Kassie on behalf of the co-authors

**POINT BY POINT RESPONSE TO THE EDITOR AND REVIEWER COMMENTS**

**RESPONSE TO ACADEMIC EDITOR:**

1. *Please ensure that your manuscript meets PLOS ONE's style requirements, including those for file naming.*

**RESPONSE: We have checked and have ensured that the manuscript met the journal’s requirements.**

2. *Please ensure that you refer to Figure 4 in your text as, if accepted, production will need this reference to link the reader to the figure*

**RESPONSE:** **Thank you for pointing out this point. We accept and revision has been made in the main document.**

*Please include captions for your supporting information files at the end of your manuscript, and update any in-text citations to match accordingly*

**RESPONSE: Thank you; we have included captions as supporting information at the end of our manuscript**

**POINT BY POINT RESPONSE TO REVIEWER**

*Well written and methodological sound manuscript. A few minor corrections:*

**RESPONSE: We thank the reviewer for kind work and constructive comments. We have revised our manuscripts based on the reviewer’s comments as below.**

*Line 45: Better to say 1 in 4 children rather than one fourth of children*

**RESPONSE: Thank you, we have replaced the term as suggested**

*Line 81: Sentence needs restructuring to read as follows:- ‘This indicates that feeding of the minimum acceptable diet is a major problem in Ethiopia. You cannot say global problem when you have only presented data for Ethiopia*

**RESPONSE: Agree, we have modified accordingly**

*Line 170: Capital letters for Egger’s and Begg*

**RESPONSE: Thanks for this comment, corrected**

*Line 191: Define cases and Prevalence in your table. What cases and prevalence of what?*

**RESPONSE: Thanks for the this suggestion, we have defined cases in the main document as “children who met Minimum Acceptable Diet (MAD)” and prevalence as “Prevalence of MAD”**

*Line 235: restructure sentence to read ‘national Demographic and Health surveys in China, ….*

**RESPONSE: Thank you for your suggestion, the sentence have restructured as “*The result of this meta-analysis was consistent with the national Demographic and Health surveys in China”***

*Line 237: remove ‘finding from’*

**RESPONSE: Thank you, noted and deleted**

*Line 238: ‘in low- and middle-income countries…*

**RESPONSE: Comment accepted and well treated**

*Line 234: heterogeneity without capital H*

**RESPONSE: Thank you, accepted**

*Line 256: Correction: higher prevalence of MAD was observed in studies conducted in urban settings compared to studies conducted in rural settings.*

**RESPONSE: Thank you, we have replaced the sentence as indicated**

*Line 265: restructure sentence to read ‘more likely to be fed the MAD standard…*’

**RESPONSE: Comment accepted and revised as recommended**

*Line 268: delete after the end of the MDGs*

**RESPONSE: We thank you again, we have edited accordingly**

*Line 268-271: This sentence does not add any value to the discussion of IYCF or MAD. Or restructure to say how the new 2019 Nutrition programme is addressing IYCF or MAD.*

**RESPONSE: Thank you for insightful comment. The sentence putted prior to this information was not related with this discussion that is why it was confused. Therefore, the indicated sentence is moved to the preceding paragraph and I hope the information from “line 268-271” add a value as a justification why MAD is higher in the studies published after the year 2020 which might be due to the implementation of new 2019 food and nutrition policy in Ethiopia aimed to achieve optimal nutritional status throughout the life cycle. We also made some restructuring of the sentence.**

*Line 277: 1 in 4 children rather than ‘one fourth of children…’*

**RESPONSE: Thank you, we have replaced the term as suggested**
