# Supplementary material for: Minimum acceptable dietary intake among children aged 6–23 months in Ethiopia: A systematic review and meta-analysis
Source: PLoS One. 2023 Jun 29;18(6):e0287247. doi: 10.1371/journal.pone.0287247 (PMC10310018; doi:10.1371/journal.pone.0287247)
Supplement: Attachment — Submitted filename: Reviewer to reviewers.docx [file pone.0287247.s005.docx]

**May 13, 2023**

**PLOSE ONE**

**PONE-D-22-14108R2: Minimum acceptable Dietary intake among children aged 6-23 months in Ethiopia: a systematic review and meta-analysis**

**Dear Academic Editor,**

Thank you for considering our revised manuscript and for arranging for it to be reviewed by reviewers. We have tried to address your comments and the comments / suggestions from the reviewer. We have highlighted the changes as track changes in the manuscript please find for your kind consideration the following: In the Response to Reviewers, we copy each of the *comments* / *suggestions* and provide the **RESPONSE** hereunder. We also provide a marked-up copy of the manuscript that highlights changes made to the original version and this is uploaded as a separate file labeled “*Revised Manuscript with Track Changes*”. Finally, we provide an unmarked version of the revised manuscript without tracked changes and this is 14 uploaded as a separate file labeled 'cleaned Manuscript'.

We have been carefully through the peer review and have revised our paper accordingly. We feel that the paper is much improved as a result of this peer review process, and thank you for taking it to this stage. We hope we have satisfactorily addressed all the comments and hope that our paper may now be suitable for publication in your journal. We hold ourselves at your entire disposition for any further information or other changes you might require..

Sincerely yours!

Gizachew Ambaw	Kassie	on behalf of the co-authors

**Point-by-point response to reviewers**

**Reviewer #2:**

**General comment:** The rationale for the present meta-analysis is rather weak and unclear. The pooled prevalence estimate is not useful or informative given the very substantial heterogeneity (I2 > 99%) among the different studies and contexts being pooled.

**Response:** Dear reviewer, we would like to express our gratitude for the tie you have taken to insightful and constructive comments on our manuscript, and we appreciate the effort you have put in to review our paper. Yes, of course high heterogeneity can affect our result. However to address this significant heterogeneity; we have estimated the pooled prevalence by random effect model. As well as we performed subgroup analysis and meta-regression to identify the possible cause of the heterogeneity. In addition, we acknowledge the limitations and uncertainties associated with meta-analyses and to interpret the findings cautiously in light of the available evidence.

Specific comments:

**Comment** 1: This is a "systematic review" and not a "Systemic Review".

**Response:** Thank you for pointing out it. We have corrected it in the revised version **Comment** 2: Please change "Even though, many study" to "Even though many studies".

**Response:** Thank you! Comment accepted and corrected as recommended.

**Comment** 3: Please change "The Global burden" to "The global burden".

**Response:** Thank you, comment accepted and well treated accordingly.

**Comment** 4: As per PRISMA guidelines, please specify in the methods section if the review protocol was prospectively registered. Indicate if a review protocol exists, if and where it can be accessed (e.g., Web address).

**Response:** Thank you again for suggestion and comment. We have stated as “the protocol is was not registered

**Comment** 5: The reliability and reproducibility of a systematic review cannot be overemphasized. Please also provide the full electronic search strategy used to identify studies, including all search terms and limits for at least one database. The rest can be provided in the supplementary material.

**Response:** Thank you again for suggestion and comment. We have included the searching terms for PubMed in the main manuscripts in addition. We have also attached the searching strategy as supplementary files.

**Comment** 6: Please change "gray literature" to "grey literature".

**Response:** Comment accepted and corrected as “grey”

**Comment** 7: Please change "Homogeneity across studies" to "Heterogeneity across the studies".

**Response:** Thank you for pointing out it. Corrected in the revised manuscript

**Comment** 8: "Full title articles excluded with reason (n=21)" - please state the exact reasons for exclusion.

**Response:** Thank you, comment accepted and well treated accordingly.

**Comment** 10: The findings of the result were not well discussed. What minimum dietary intake value do the authors consider to be acceptable, considering the characteristics of the country, the population and the environment? What specific recommendations can be made to address these gaps?

**Response:** Thank you! We have added limitation regarding heterogeneity

**Comment** 11: The discussion of study limitations was grossly inadequate. How can we account for the very significant heterogeneity in the meta-analysis? Equally if not more important than the point estimate, the distribution of estimates (i.e., how much they are dispersed around the average pooled estimate) is a key aspect in meta-analyses of prevalence. Are the studies truly comparable?

**Response:** Thank you, we have made extensive revision

**Comment** 12: "We would like to thank all of the authors of studies participated in this systematic review and meta-analysis" - this does not make sense. Suggest to omit.

**Response:** Thank you pointing out this error. We have omit it based on the comment

**Reviewer #3:**

General comments

**Comment** 1: The work lacks nobility. Since there is EDHS 2019 that reported a prevalence of 11% minimum acceptable diet, it is not possible to generate a pooled prevalence that is more valid and reliable than the EDHS 2019 finding. How can your finding be preferred over EDHS 2019 findings? Your pooled prevalence is very far to represent the national prevalence as the included studies are few and obtained from only four regions out of a total of 11 regions in the country.

**Response:** Thank you reviewer for devoting your time to review our manuscript and your comments and suggestions. We have corrected it in the revised version of the manuscript. We have stated this issue as a limitation.

**Comment** 2: I suggest a systematic review only. You should not pool prevalence (never conduct a meta-analysis).

**Response:** Thank you very much for you suggestions. Even though, some limitation of this meta-analysis, we believe that our finding has a greatest implication for policy maker because meta-analysis increase the power of the study

**Comment** 3: there is a serious English language editorial problem

**Response:** Thank you, we have checked grammatically errors, writing style and typos; and made extensive revisions in the revised manuscript.
Specific comments
Abstract
Introduction:
**Comment**: Line 27: remove “an adequate” as “minimum acceptable diet” is by itself enough to convey the intended message.

**Response:** Thank you for pointing out this comment. We have revised it accordingly in the revised manuscript
**Comment**: Line 28 and second sentence: It says “Feeding the minimum acceptable…………” Correct as “Not feeding at least the minimum acceptable diet…….’’

**Response:** Thank you for pointing out it. Corrected based on the comment **Comment**: Line 30: Your objective was to produce a pooled prevalence as a result of a rationale of inconsistence studies in Ethiopia. The problem you actually observed from your result is a serious consistence across studies as evidenced by your heterogeneity test. So, I recommend you end up with systematic review only rather than pooling a very heterogeneous results which may be due to small number of studies.

**Response:** Thank you for bringing this issue. To address these limitations, we carefully assess the sources of heterogeneity, conduct subgroup analyses, use appropriate statistical methods, and report the results transparently and comprehensively. We have acknowledge the limitations and uncertainties associated with meta-analyses and to interpret the findings cautiously in light of the available evidence

Method:
**Comment**: Line 35: “Data was” correct as “Data were”

**Response:** Thank you pointing out the error. We have corrected it
Results:
**Comment**: What was the source of heterogeneity since you mentioned in the method section above that you did a subgroup analysis to identify the source of heterogeneity?

**Response:** Thank you for your question. The observed high heterogeneity might be due to the difference in the study area, study period, residence and other unspecified variations.

**Comment**: Key words (incorrect), Keyword (correct): Separate the keywords by a semicolon not by a comma.

**Response: thank you we have revised it**

**Comment**: As there was 2019 EDHS’s report of 11% minimum acceptable diet proportion, how strong your finding will be in attracting the governments attention as compared to the EDHS 2019 finding? Due to this and other reasons, I suggest you simply conduct systematic review only.

**Response:** Dear reviewer, we would like to thank you for your suggestions. However, despite searching different databases, we were unable to find systematic reviews or meta-analyses on the minimum acceptable diet. Therefore, the papers reviewed were discussed with those of the national studies

**Comment**: Line108 and 109: Published and unpublished reports: There should be consistency. Which one you really did in your review? Only published (in the abstract) or both Published and Unpublished (introduction).

**Response:** we apologize for the inconvenience. We have corrected it based on your comment
**Comment**: Line 116: Which searching terms were combined by "and" which were combined by "or"?

**Response:** Comment accepted, we have revised it in the revised draft
**Comment**: Line 127: Exclusion criteria – not published in English was already mention in the inclusion criteria.

**Response:** Thank you for the comment. We included articles published only in English language for the sake of clarity, understandability, and simplicity of interpretations. In addition, we have stated as a limitation  **Comment**: Line 137: What is that standardized data abstraction sheet you used?

**Response:** We used excel spreadsheet to extract the data
Results
**Comment**: The nine studies are all from three regions and one city administration. So, meta-analysis is not important.

**Response:** Thank you for your suggestion.
**Comment**: Line 191: Remove meta-analysis in the bracket.

**Response:** Thank you, we have removed it
Discussion
**Comment**: Line 232: Please cite for the global recommendations at the end of “This Indicates that most children aged 6–23 months in Ethiopia were not taking the minimum acceptable diet according to global recommendations”

**Response:** than you we have cited appropriately.
**Comment**: Line 234-235: You compared your finding with other Countries’ DHS report. So, the Ethiopian Demographic and Health Survey finding is more comparable with those DHS reports than your pooled prevalence. So, I recommend only systematic review.

**Response:** Despite searching different databases, we were unable to find systematic reviews or meta-analyses on the minimum acceptable diet. Therefore, the papers reviewed were discussed with those of the national studies

**Comment**: Line 236: You again compared your finding with the EDHS 2019 report and yours was higher. The explanation you gave for the difference was your study was not representative of all regions as EDHS was. So, this shows your study was not important at all. Because, the EDHS finding is valid than yours

**Response:** Thank you very much for your interesting comments and suggestions. One important thing for a research is creating a question for other researchers in order to conduct further research on that specific problem. To the best of my knowledge, it is the first meta-analysis and the finding too. Hence, we give a justification about its discrepancy. In addition, we have mention the problem in the limitation.
Limitation and strength
**Comment**: Line 271: Why you only included English language articles?

**Response:** Thank you for the comment. We included articles published only in English language for the sake of clarity, understandability, and simplicity of interpretations.
**Comment**: Line 272: Since us already the EDHS 2019 report that has not the limitations encountered by your study, EDHS is more important than yours.

**Response:** dear reviewer thank you for your comment. As we know one of the key characteristics of research is a single study cannot stand alone. We need to have different research finding with different approaches in order to implement appropriate intervention mechanisms
Conclusion and recommendation
**Comment**: “The result of this systemic review and meta- analysis indicated that minimum acceptable diet among children aged 6-23 months was found to be low in Ethiopia; only 1 in 4children met the minimum acceptable diet.” Your study finding (25%) is better than EDHS finding (11%). So, your recommendation is not acceptable since it has serious limitations.

**Response:** Thank you. We compare our finding based on the WHO and other guideline recommendation. The World Health Organization (WHO) recommends ensuring a minimum acceptable diet (MAD) for all children, particularly in the early years, to promote healthy growth and development

References
**Comment**: References are not edited and formatted

**Response:** Thank you, we have edited and we have corrected the formats based on your comment
